# Regional Prevalence of Hemoglobin C Across Saudi Arabia: An Epidemiological Survey

**DOI:** 10.1007/s44197-024-00193-w

**Published:** 2024-02-19

**Authors:** Mansour Aljabry, Suha Sulimani, Ghazi Alotaibi, Hassan Aljabri, Shaker Alomary, Izzeldin Adam, Omar Aljabri, Mansour Khater Alzahrani, Abdulrahman Alsultan

**Affiliations:** 1 Pathology Department, College of Medicine, King Saud University, Riyadh, Saudi Arabia; 2 Premarital Program, Ministry of Health, Riyadh, Saudi Arabia; 3 Department of Medicine, College of Medicine, King Saud University, Riyadh, Saudi Arabia; 4 Laboratory Department, Cluster 3 in Riyadh Region, Ministry of Health, Riyadh, Saudi Arabia; 5 Family Medicine Department, College of Medicine, Majmaah University, Al Majma’ah, Saudi Arabia; 6 Department of Pediatrics, College of Medicine, King Saud University, Riyadh, Saudi Arabia

**Keywords:** Hemoglobin C, Hemoglobinopathies, Saudi Arabia, Genetic counseling, Pre-marital screening, Prevalence

## Abstract

**Introduction:**

HbC is a common structural hemoglobinopathy especially in West Africa. Prevalence and regional distribution of HbC in Saudi Arabia are widely undocumented. Patients with homozygous HbC disease may have mild hemolytic anemia whereas combination with hemoglobin S (HbS) leads to a clinically severe phenotype.

**Aim:**

The current epidemiological study, considered the largest from Saudi Arabia, aimed to evaluate the regional prevalence of the HbC variant among the couples participating in the premarital screening program from 2011 to 2018.

**Methods:**

Data from the PMSGC program were obtained for premarital screening and genetic counseling. The collected data were then entered into the SEHA platform, a centralized electronic repository for the 13 designated regions in Saudi Arabia. Hemoglobin electrophoresis samples are analyzed using either HPLC, capillary electrophoresis, or a combination of both methods to confirm the presence of abnormal hemoglobin bands.

**Results:**

This study included 1,871,184 individuals from 2011 to 2018. Of those, 49.8% were males and 50.2% were females. 112,618 (6.0%) had an abnormal test. Total number of Hb C cases were 778 (0.04%). HbC trait (HbAC) was detected in 764 participants while homozygous HbC (HbCC) and combined heterozygous (HbSC) were found in 9 and 5 cases, respectively. The regions near the Red Sea have higher rates than the central and eastern regions.

**Conclusion:**

HbC is a rare variant in Saudi Arabia with varying regional frequencies. HbC variant is more common in Mecca and Madina regions. The geographic area of HbC distribution differs from the areas with high prevalence of HbS, which explains why HbSC disease cases are overwhelmingly rare.

## Introduction

Structural hemoglobinopathies encompass a wide variety of hemoglobin disorders caused by changes in the globin genes that can, in turn, alter the overall structure of the globin [1]. Unlike thalassemia, the pathogenesis of structural hemoglobinopathies results from the altered physical properties of the abnormal hemoglobin. These changes might manifest as reduced solubility, altered oxygen affinity, reduced protein stability, or methemoglobinemia [2].

Hemoglobin C (HbC) is an abnormal hemoglobin in which a lysine residue is substituted for glutamic acid at the sixth position of the beta (β)-globin chain. This substitution is caused by a point mutation in the hemoglobin β (HBB) gene [3]. Individuals with hemoglobin C trait (Hb AC) are generally asymptomatic and phenotypically normal, but carriers can transmit the defective gene to their progeny in an autosomal recessive inheritance pattern [4]. On the contrary, patients with homozygous HbC disease (Hb CC) may present with chronic compensated hemolysis, jaundice, splenomegaly, and gallstones. Similarly, combined heterozygous for HbC with hemoglobin S (Hemoglobin SC disease) leads to sickling disorder, which is usually less severe than homozygous sickle cell anemia (SCA) [4]. Retinal disease, splenic crises, and avascular necrosis are more common in hemoglobin SC disease than SCA. Hemoglobin level is higher than that of SCA, ranging from 8 to 12 g/dL with normal mean cell volume (MCV) and mean cell hemoglobin (MCH), whereas mean cell hemoglobin concentration MCHC is high due to hypodensity and presence of HbC crystals [5, 6].

HbC is a common structural hemoglobin variant, especially in West African countries and South East Asia, correlating with the prevalence of malaria infection as it exerts a protective effect against the malaria parasite [7]. Sporadic cases of HbC trait or disease have been reported in a diverse population in the Middle East, North Africa, Southern Europe, and South and Central America [8]. However, the spread of hemoglobin C outside Atlantic West Africa and Southeast Asia is widely assumed to be related to immigration and international marriages [9].

Prevalence and regional distribution of HbC in Saudi Arabia are widely undocumented and obtained mainly from small case series or case reports [10, 11]. Yet, such data are necessary to estimate the burden of HbC in public health and its contribution towards sickle cell disorders alongside hemoglobin S.

In 2004, the premarital screening and genetic counseling (PMSGC) program was implemented as a compulsory program for all the engaged couples [12]. The PMSGC program was specifically designed to detect thalassemia and sickle cell anemia through hemoglobin electrophoresis, complete blood counts (CBC), and iron profile studies [13]. Besides hemoglobin S, the program includes detecting some beta-hemoglobin variants such as HbC, hemoglobin D, and Hemoglobin O-Arab from the 13 designated administrative regions in Saudi Arabia [12].

The current study aims to evaluate the prevalence of HbC and its pattern of regional distribution across Saudi Arabia, utilizing the data from the national premarital and genetic counseling program from 2011 to 2018.

## Methods

### Study Design and Participants

The present epidemiological study is a retrospective research obtained the data from the PMSGC program, which collects information from individuals who underwent genetic counseling and premarital screening. The data for the genetic counseling and premarital screening performed from 2011 to 2018 were included in this study. The collected data were then entered into the SEHA platform, a centralized electronic repository for the 13 designated regions in Saudi Arabia. The key parameters included the patient gender, region/area of enrollment, and the presence and frequency of hemoglobin variant HbC.

This study received approval from the Institutional Review Board of the Ministry of Health, Saudi Arabia (IRB log No: 23-18 E). The study was designed as a non-interventional study; therefore, informed consent was not obtained from the participants. Each participant was allotted an exclusive identification code, and the data were stored in the electronic record systems using this identification code to ensure the subject data privacy.

### Hemoglobinopathies Testing

The EDTA samples were obtained from the relevant authorities. The hospital's central reference laboratory processed the research samples. Before being examined in a laboratory, these samples were stored at ambient temperature. All the blood samples underwent a CBC. Using an automated blood counter (Sysmex XE-2100; Sysmex Corporation, Kobe, Japan),) MCH and MCV were determined.

Hemoglobin electrophoresis samples were analyzed using either HPLC, capillary electrophoresis, or a combination of both methods to confirm the presence of abnormal hemoglobin bands. Following the manufacturer's instructions, Hb was separated using HPLC (VARIANT II; Bio-Rad Laboratories, Hercules, CA, USA) and CE (CAPILLARYS 2; Sebia, France) techniques. The two techniques separately analyzed samples within 12 h. Version 6.12 of the Sebia CAPILLARYS 2 (Phoresis) software was used for fraction identification and Hb quantification.

### Data Analysis

Under the direct supervision of the general directorate of health affairs, regional premarital centers collected and managed the data. The Saudi Central Board has accredited all Accreditation of Healthcare Institutions (CBAHI) laboratory centers. The physician assigned to each premarital center must authorize the results after the laboratory supervisors enter the information into the Seha Platform database.

For the concluding statistical analysis, data from the Seha platform were extracted and organized in an Excel file. The frequency of uncommon hemoglobin variants was expressed per 1000 test subjects.

### Statistical Analysis

Descriptive statistics were generated for continuous data, including means, maximum and minimum values along with 95% confidence intervals (CIs), and standard deviations (SDs). There were cross-tabulations of categorical variables and frequency of occurrence. MATLAB v.2023a (Mathworks Inc, Natick, MA, USA) was utilized to conduct the fundamental statistical analysis on the included individuals from multiple Seha platform data sources using MATLAB.

## Results

This study included 1,871,184 individuals from 2011 to 2018, Table 1 of those, 49.8% (*n* = 931,099) were males and 50.2% (*n* = 940,085) were females. The study cohort's mean age (± SD) was 30.2 ± 8.0 years. Total number of HbC cases were 778. The mean value of hemoglobin (± SD) was 13.5 ± 2.0 g/L. The mean RBC (± SD) count was 5.3 ± 0.7 × 10^12^. Mean MCV (± SD) was calculated as 76.3 ± 8.4 fL, and mean MCH (± SD) was detected to be 25.6 ± 3.5. The mean HbF value was found to be 0.55%. (Table 2).

**Table 1:** The premarital screening program between 2011 and 2018 by gender in Saudi Arabia

	Male	Female	Total
Number of Tested Individuals	931,099	940,085	1,871,184
Mean Age in years (± SD)	32.8 (± 7)	27.5 (± 7)	30.2 ± 8.0
HbC			
HbCA	392	372	764
HbCC	5	4	9
HbSC	3	2	5
Total	400	378	778 (0.04%)

**Table 2:** The mean values and standard deviation of red cell indices and Hb F for the Hb C. variant

	No	Mean Hb % ± SD	Mean RBC ± SD (× 10^12^ /L)	Mean Hb ± SD (g/L)	Mean MCV ± SD (fL)	Mean MCH ± SD (pg)	Mean HbF (%)
Male	400	30 ± 12.7	5.6 ± 0.6	14.9 ± 2.4	79.5 ± 3.9	27 ± 4.1	0.57
Female	378	28.1 ± 9.8	4.9 ± 0.7	12.7 ± 1.9	72.9 ± 5.4	24.1 ± 5.2	0.53
Total	778	29.0 ± 14.5	5.3 ± 0.7	13.5 ± 2.0	76.3 ± 8.4	25.6 ± 3.5	0.55

The mean age of the male participants was 32.8 years, and that of female participants was found to be 27.5 years. The total number of abnormal tests in the entire cohort was 112,618 (6%) participants. 392 male participants were found to be Hb CA, 5 were detected as Hb CC, and three were detected as Hb SC. 372 female participants were Hb CA, 4 participants were Hb CC, and only 2 females were Hb SC. The total detection of Hb C out of all participants was 778 (0.04%) (Table 1).

**Fig. 1:**
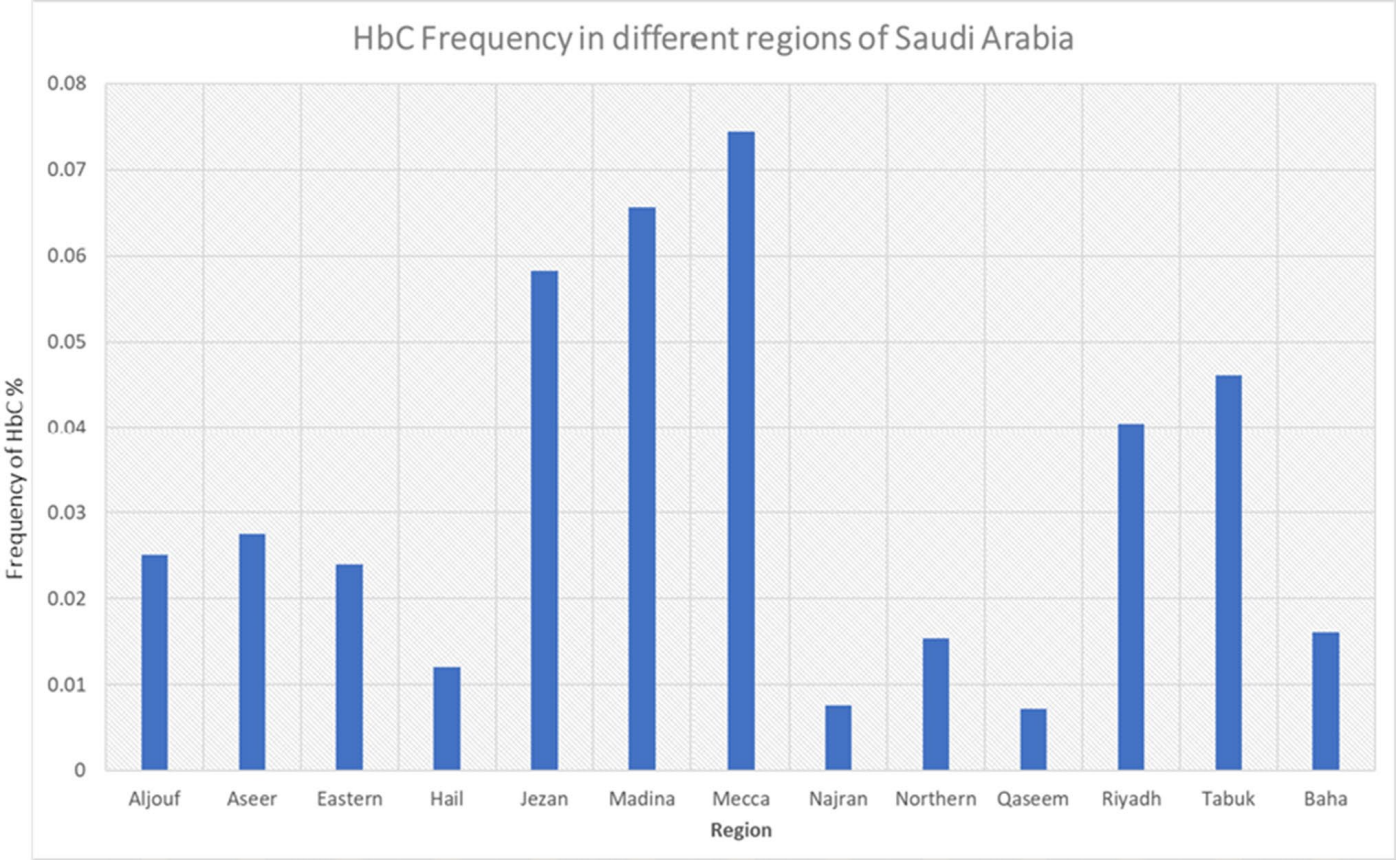
Graphical depiction of the frequency of HbC in different regions of Saudi Arabia

**Fig. 2:**
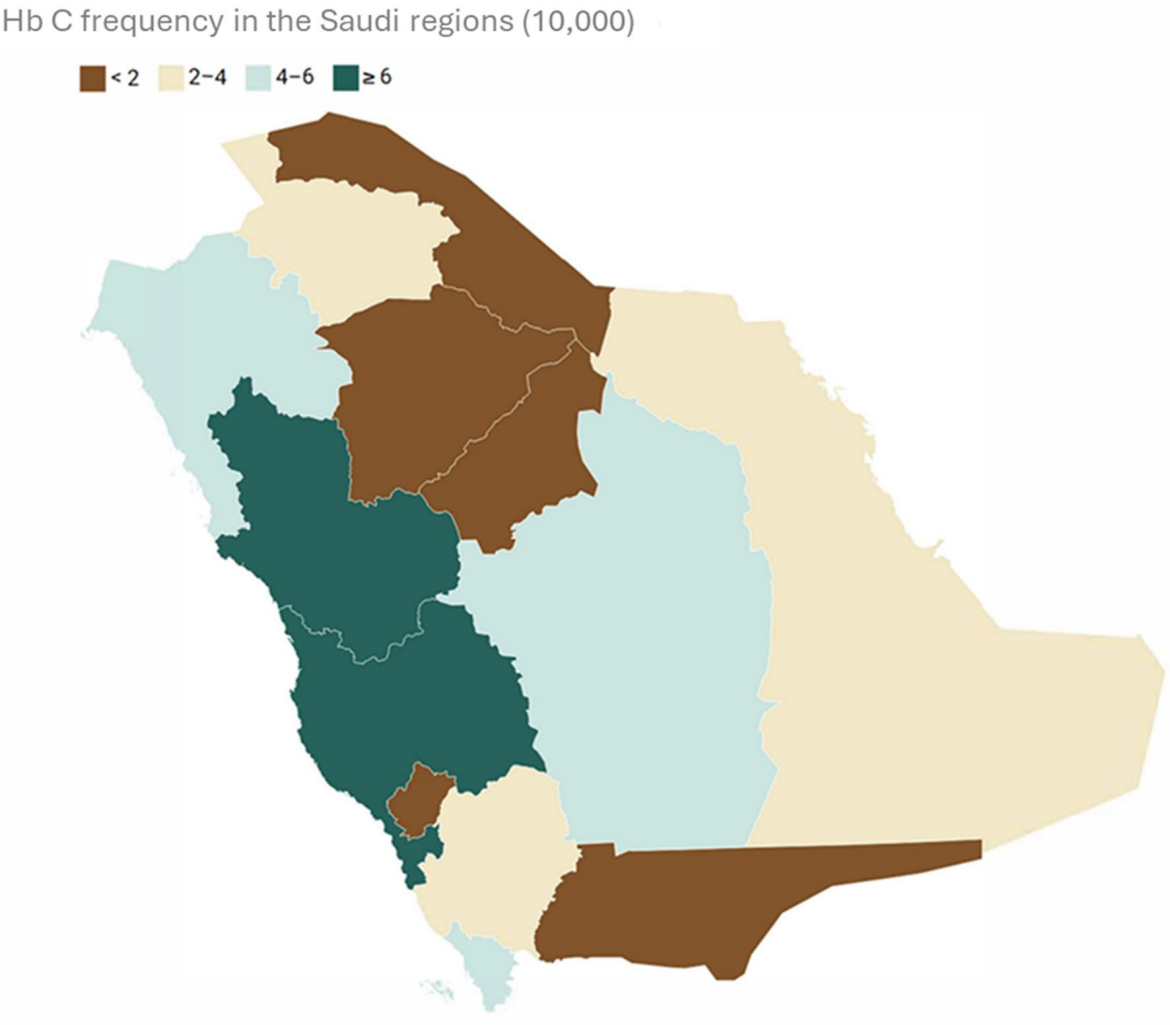
Frequency of HbC depicted in Saudi Arabia Map

The total number of tests performed in Aljouf region was 31,855. Of these, 8 cases were HbC positive. In Aseer and Eastern region, 228,908 and 325,758 tests were performed. Of these, 60 and 78 cases were positive, respectively. In Jezan, 121,784 tests were performed, and 71 were found positive for HbC. 7 out of 58,146 cases were positive for HbC in the Hail region. In Madina and Mecca, 87 and 290 cases were found positive from the 132,532 and 389,475 tested, respectively. In Najran and Baha, 3 each were found positive out of the 39,683 and 18,650 tests performed, respectively. In Riyadh, 126 tests were found positive out of 311,618 tests performed. In Tabuk, 32 tests were found positive out of 69,448 tests performed. In the Northern region, 5 tests were positive out of 32,575 tests performed. In Qaseem, 8 tests were positive from 110,752 tests. The frequency of distribution is depicted in Fig. 1 and Fig. 2.

## Discussion

The current study represents the largest data for HbC prevalence patterns across Saudi Arabia. The study revealed that the overall prevalence of HbC is 0.04% of all tested individuals with marked regional variation. The frequency of HbC in Saudi Arabia is relatively low compared with data obtained from West African countries, African American countries, and Brazil [14, 15]. The epidemiological studies have determined the maximum frequency of HbC in the eastern region of Burkina Faso, ranging from 12 to 21%, with a median frequency of 16% across the entire population. This "hot spot" is surrounded by a wider circle, which includes all West African countries, with a prevalence rate of 5–11% [16]. According to Piel et al*.*, the frequency of HbC extends across East and North Africa with a progressive decline until it reaches 1% in Egypt [14]. As for the eastern coasts of the North and South American continents, the percentage ranges between 1 and 3%, involving mainly African-American regions [17].

The HbC variant was detected across all the regions of Saudi Arabia; however, the prevalence of the variant varied significantly from region to region. The highest frequency was discovered across the coast of the Red Sea in Mecca and Madina regions, with 0.075% and 0.065%, respectively. This can be attributed to a large community of African descent inhibiting the two holy cities and the increasing number of West African pilgrims visiting Mecca and Madina for Hajj and Umrah. Hawsawi et al. reported two cases of Hemoglobin SC disease in Madina regions from West African ancestry. One of the patients had mild disease, whereas the other had severe features, including transient hypertension [18].

The HbC allele is less common in the Najran and Qassim regions of Saudi Arabia (less than 0.01% of the population) but relatively more common in the Jizan and Tabuk regions (0.055% and 0.045% of the population, respectively). This is likely because Jizan and Tabuk are located on the Red Sea coast, which has historically been a major trade and pilgrims' route between Africa and the two holy cities.

The Eastern region of Saudi Arabia is known to have the highest prevalence of hemoglobinopathies, mainly of sickle cell disease and thalassemia. In 1976, two cases of HbC trait were reported from the Eastern region in a screening program that included 391 apparently healthy adult males [19]. To our knowledge, no cases of HbC have been reported from the Eastern region following that study. The current study has revealed a low prevalence of HbC in the Eastern region compared to the Mecca and Madina regions.

The vast majority of HbC cases in our study were heterozygous (Hb CA), whereas homozygous Hb CC and combined heterozygous Hb SC disease have been detected in about 2% of the participants, only representing nine and five cases, respectively. HbSC is of clinical significance owing to the increased risk of sickle cell crises, thrombotic events, and splenic crises. Of note, the geographic area of HbC distribution differs from HbS in Saudi Arabia, which might explain the low prevalence rates.

A retrospective analysis of 111 HbC cases in Morocco revealed higher percentages of homozygous Hb CC disease and combined heterozygous HbSC with 8% and 9% frequencies, respectively [20]. HbC trait was detected in 75%, and HbC/O-Arab in 2% of cases [16].

The present study also estimated the impact of HbC on hematological indices. Hemoglobin values, RBC count, and hemoglobin F levels are relatively close to the normal ranges detected in the healthy adult Saudi population. However, MCV and MCH levels are lower than the index values [21]. This observation can be related to a high prevalence of latent iron deficiency and alpha thalassemia in the Saudi population.

## Conclusion

The present study is the largest on the prevalence of HbC variant in the Saudi population and identified that HbC is a rare variant in the Saudi population. However, the incidence has been higher in the regions with the pilgrims’ influx and the expatriate populace. The geographic area of HbC distribution differs from HbS in Saudi Arabia, which explains why HbSC disease cases are overwhelmingly rare. Further molecular studies utilizing high throughput technology are recommended to elucidate the genetic landscape of HbC variant in the Saudi population.

## Data Availability

Furnished upon reasonable request to the corresponding author.

## Abbreviations

PMSGC: Premarital screening and genetic counseling program.
CBAHI: Saudi Central Board has accredited all Accreditation of Healthcare Institutions
HbAC: HbC trait
HbCC: Homozygous HbC
HbSC: Combined heterozygous of Hemoglobin S &hemoglobin C
